# Predictors of Willingness to Receive the COVID-19 Vaccine after Emergency Use Authorization: The Role of Coping Appraisal

**DOI:** 10.3390/vaccines9090967

**Published:** 2021-08-29

**Authors:** Qianyi Xiao, Xin Liu, Ruru Wang, Yimeng Mao, Hao Chen, Xiaomei Li, Xiaoxi Liu, Junming Dai, Junling Gao, Hua Fu, Pinpin Zheng

**Affiliations:** Department of Preventive Medicine and Health Education, School of Public Health, Fudan University, Shanghai 200032, China; xiaoqianyi@fudan.edu.cn (Q.X.); 19211020043@fudan.edu.cn (X.L.); 18211020093@fudan.edu.cn (R.W.); 18211020031@fudan.edu.cn (Y.M.); 18111020014@fudan.edu.cn (H.C.); 18211020030@fudan.edu.cn (X.L.); 19211020041@fudan.edu.cn (X.L.); jmdai@fudan.edu.cn (J.D.); jlgao@fudan.edu.cn (J.G.); hfu@fudan.edu.cn (H.F.)

**Keywords:** COVID-19 vaccine, willingness, emergency use authorization, protection motivation theory, China

## Abstract

The current study aims to identify psychosocial factors based on protection motivation theory (PMT) influencing Chinese adults’ willingness to receive the COVID-19 vaccine after the emergency use authorization of the New Coronavirus Inactivated Vaccine in China. A cross-sectional online survey was conducted among Chinese residents aged 18–59 years, and 2528 respondents from 31 provinces and autonomous regions were included in the current study. Based on PMT, threat appraisals and coping appraisals were measured. Hierarchical multiple regressions and multivariate logistic regressions were used to identify the relationships between the PMT constructs and vaccination willingness after other covariates were controlled for. A total of 1411 (55.8%) respondents reported being willing to receive the COVID-19 vaccine. The PMT model explained 26.6% (*p* < 0.001) of the variance in the vaccine willingness. The coping appraisals, including response efficacy, self-efficacy, and response costs, were significantly correlated with the willingness to receive the COVID-19 vaccine, and response efficacy was the strongest influencing factor (adjusted OR = 2.93, 95% CI: 2.42–3.54). In conclusion, the coping appraisals for vaccination, instead of threat appraisals regarding the pandemic itself, mainly influenced people’s willingness to get vaccinated after the emergency use authorization of the COVID-19 vaccine in China. These findings are helpful for developing education and interventions to promote vaccination willingness and enhance public health outcomes during a pandemic.

## 1. Introduction

The world is in the midst of the COVID-19 pandemic. As of 8 June 2021, more than 173 million cases of COVID-19 infections and more than 3 million deaths had been reported globally [1]. Vaccination is a pivotal means to help prevent COVID-19 and subsequent death and to control the spread of this pandemic. As of 8 June 2021, at least 13 different vaccines across four platforms have been administered in countries, and 7 vaccines have been listed for the World Health Organization (WHO) Emergency Use Listing (EUL) [2]. On 30 December 2020, the China National Medical Products Administration (CNMPA) approved the New Coronavirus Inactivated Vaccine (Vero Cell) against COVID-19 for emergency use authorization for adults aged 18 to 59 in China [3]. However, in the early stage of the vaccine rollout, there have been concerns about the vaccination rates. For effective vaccines, the inadequate use of the vaccine can become a public health issue, as declining vaccination rates at the population level could lead to an increase in morbidity and mortality from diseases that are vaccine-preventable [4,5]. Therefore, understanding the determinants of the willingness to obtain the COVID-19 vaccine is necessary, especially in the early stage of vaccine approval, to inform future vaccination programs and campaigns and to increase the uptake of the COVID-19 vaccine in China.

Vaccines are an important milestone and an ideal contribution towards strong declines in both the incidence rate and mortality rate of many infectious diseases [6,7]. However, despite these known benefits, every time a new vaccine is introduced, there is always an unwillingness and opposition to the vaccination due to some levels of fear and barriers by the public [8,9]. Previous studies have indicated that psychosocial factors based on the Health Belief Model and the Theory of Planned Behavior may increase the intention/willingness to receive the COVID-19 vaccine [10,11].

However, the current evidence on the influencing factors of receiving the COVID-19 vaccine is limited by the survey period occurring before the vaccine was approved for marketing in China, which failed to take into account the impact of whether the vaccine truly existed on the association between psychosocial factors and vaccination willingness.

Protection motivation theory (PMT), formulated by Ronald Rogers [12,13] and Richard Lazarus [14], is often used to explain whether and how people take precautionary measures against communicable diseases. PMT has been successful in predicting hepatitis B vaccination intention and behavior among an adult population in China [15]. The core of PMT is that the influence on a person’s protection motivation is grouped into two main categories: threat appraisal and coping appraisal. Threat appraisal subsumes beliefs about the severity of the threat to health and about a person’s susceptibility or vulnerability to the threat. Coping appraisal subsumes beliefs about how efficacious protective behaviors can possibly be, which are defined as response efficacy, self-efficacy (the perception of ability to perform the protective behavior), and the response cost of protective measures [16,17], which comprise unintended and possibly harmful side effects. For coping appraisals, strong positive associations between the response efficacy (perceived effectiveness) of the vaccination and the intention/willingness to be vaccinated have been found [18,19,20,21,22]. In the early stage of the emergency use authorization for the COVID-19 vaccine, the coping appraisal of the vaccination is a direct factor for the willingness to obtain the COVID-19 vaccine. Self-efficacy and perceived behavioral control (the equivalent of self-efficacy) have also been found to predict vaccination behavior [23]. In the case of preventable communicable diseases, PMT postulates that a person will be more motivated to get vaccinated, the more alarming their threat appraisals and the more promising their coping appraisals are.

Based on PMT, this study first investigates the psychosocial factors influencing vaccination willingness in the early stage of the emergency use authorization for the COVID-19 vaccine and aims to provide ways to promote vaccination willingness among the public and a reference for epidemic control in other countries.

## 2. Materials and Methods

### 2.1. Survey Design, Setting, Participants, and Recruitment

A cross-sectional, web-based anonymous survey was performed among Chinese residents living in China using an online questionnaire, which was made available on the Wenjuanxing platform (https://www.wjx.cn/app/survey.aspx, accessed on 1 January 2021) between 1 and 24 January 2021. The online survey link was disseminated via websites and WeChat, on which personal information and public websites can be shared with family members, friends, and colleagues and forwarded to others by voluntary participants. The survey provided informed consent on the first page of the survey and a small monetary reward (RMB 5) after completing the questionnaire authentically. The survey was structured as a full-length questionnaire and included 54 items designed by our research team; completing the survey took approximately an average of 5 to 10 min. Before the release of the formal survey, we performed a pilot test and set 3 min as the cut-off value to exclude the invalid questionnaires of participants who were not serious about participating in the survey and just wanted the monetary reward. Additionally, a quality control item with a required answer was set to avoid the return of invalid questionnaires. Finally, a total of 2580 participants from 31 provinces and autonomous regions (China consists of a total of 34 provinces and autonomous regions) completed the survey, of whom 98.0% (*n* = 2528) met the eligibility criteria (aged 18–59 years with valid questionnaires). The public was not involved in the design, conduct, reporting, or dissemination plans of our research.

### 2.2. Measures

#### Survey Instruments

The survey consisted of questions that assessed (1) demographic characteristics, (2) willingness to receive the COVID-19 vaccination, and (3) PMT constructs. The survey did not provide information on vaccine safety and efficacy.

(1) Demographic Characteristics and Health Status

The demographic characteristics consisted of age, gender, region, marital status, education status, occupation, and average monthly income. Health status included a self-rated health status and the self-reported presence of a chronic disease ever diagnosed by doctors. Self-rated health status was measured using one question (“How is your perceived health in general?”) followed by a series of five semantic differential scales: excellent, very good, good, general, and bad [24]. Participants with answers of “excellent”, “very good”, and “good” were categorized as overall “good” in regard to self-rated health. Chronic diseases, including 11 common chronic diseases such as hypertension, diabetes, coronary heart disease, anaphylactic disease, gastrointestinal disease, and so on, were classified into three categories (0, 1–2, ≥3).

(2) Willingness to Receive the COVID-19 Vaccine

The willingness to receive the COVID-19 vaccine was a self-rated assessment measured using one question (“How willing would you be to get the COVID-19 vaccine?”) that was answered on a seven-point scale (refuse all, refuse but unsure, refuse some, delay, accept some, accept but unsure, accept all) [25]. The willingness to receive the COVID-19 vaccine was analyzed by both a continuous variable (1–7 score) and a categorical variable that was defined as two categories (1: accept all and accept but unsure; 0: all other answers).

(3) Protection Motivation Theory (PMT) Constructs

A Likert-style scale was used to measure all the items related to the PMT constructs, with a range from 1 = strongly disagree to 5 = strongly agree. The mean of all items in each construct was calculated as an overall measure of each construct. The median of the respondents’ averaged index was used for binary categorical classification (high/low level). Detailed information on the survey questions, variable description, and processing are shown in Table 1. Exploratory factor analysis (EFA) was first applied to assess the underlying structure of the items referring to PMT, and their factor loadings were in accordance with the items that were grouped into PMT constructs (Table 1).

Perceived severity. The perceived severity scale consisted of three items. A sample item is “I feel that COVID-19 is a serious infection that is harmful to my health.” For the perceived severity construct, the median and mean (SD) of the respondents’ averaged index were 4.33 and 4.06 (0.89), respectively. This measure showed good internal reliability with a Cronbach’s alpha of 0.80 [26].

Perceived susceptibility. The perceived severity scale consisted of three items. A sample item is “I am vulnerable to being infected with COVID-19.” For the perceived susceptibility construct, the median and mean (SD) of the respondents’ averaged index were 2.00 and 1.92 (0.83), respectively. This measure showed good internal reliability with a Cronbach’s alpha of 0.86 [26].

Response efficacy. The response efficacy scale consisted of three items. A sample item is “Being vaccinated against COVID-19 would be extremely effective in protecting me against COVID-19”. For the response efficacy construct, the median and mean (SD) of the respondents’ averaged index were 4.00 and 4.02 (0.76), respectively. This measure showed good internal reliability with a Cronbach’s alpha of 0.83 [26].

Self-efficacy. The self-efficacy scale consisted of three items. A sample item is “I believe that I can get the COVID-19 vaccination easily and successfully”. For the self-efficacy construct, the median and mean (SD) of the respondents’ averaged index were 3.67 and 3.63 (0.81), respectively. This measure showed acceptable internal reliability with a Cronbach’s alpha of 0.73 [26].

Response costs. The response costs scale consisted of three items that mainly focused on the body’s reaction to the behavior of the vaccination. A sample item is “Getting the COVID-19 vaccine would be inconvenient for me”. For the response cost construct, the median and mean (SD) of the respondents’ averaged index were 3.00 and 3.16 (0.89), respectively. This measure showed acceptable internal reliability with a Cronbach’s alpha of 0.69 [26].

### 2.3. Statistical Analysis

The frequencies and proportions were first calculated for the demographic characteristics according to the willingness to obtain the COVID-19 vaccine. Comparisons were conducted using the chi-square test for the categorical variables between groups of vaccination willingness. The factor loadings of items were assessed by using the exploratory factor analysis (EFA). EFA utilized a principal component analysis framework with Varimax rotation. Cronbach’s alpha was used to estimate the internal reliability of the items for study measures. Then, hierarchical multiple regression equations were set up to test the PMT model and examine the contribution of PMT in predicting vaccination willingness. Demographic variables were entered in Block 1, and PMT-related variables were entered in Block 2. The effects of the independent variables were expressed in terms of standardized regression coefficients (betas). The amount of variance explained in the model was reported in terms of R^2^. Next, multivariable logistic regression analyses were applied to determine the independent psychosocial factors associated with vaccination willingness. Covariates include age, sex, occupation, region, self-rated overall health, and the number of chronic diseases. Odds ratios (ORs) and 95% confidence intervals (CIs) were used to quantify the effects. Finally, the impact of specific psychosocial factors influencing the willingness to receive the COVID-19 vaccine was examined using multivariable logistic regression analyses. All of the analyses were carried out using SPSS software V.22.0 (SPSS, Chicago, IL, USA). All tests were two-tailed with a significance level of *p* < 0.05.

## 3. Results

### 3.1. Descriptive Statistics of the Willingness to Receive the COVID-19 Vaccine

Among the 2580 questionnaires returned, 2528 (98.0%) were valid and included in the analysis. Table 2 displays the characteristics of the 2528 survey respondents. Of all the respondents, 58.7% (*n* = 1484) were women, 75.8% (*n* = 1916) were aged 18–39 years, and the average age was 33.9 years. The majority of the respondents were married (65.6%, *n* = 1658), had a bachelor’s degree (63.6%, *n* = 1607), and lived in urban areas (89.4%, *n* = 2260). Only 19.7% (*n* = 497) were medical staff. Approximately 79.8% (*n* = 2018) had self-rated good health, and 63.9% (*n* = 1616) reported having no chronic diseases.

For the willingness to receive the COVID-19 vaccine, 1411 (55.8%) of all the respondents were willing to receive the vaccine, while 1117 (44.2%) were unwilling to receive the vaccine. The frequency between “willing” and “unwilling” respondents differed (*p* < 0.05) by age, ranging from 55.5% for those aged 18–29 years, 53.1% for those aged 30–39 years, 60.2% for those aged 40–49 years, and 61.4% for those aged 50–59 years. The frequency differed by gender, with a higher frequency in men (59.7%) than in women (53.1%). The frequency differed by occupation, with a higher frequency in medical staff (48.7%) than in non-medical staff (7.5%); it also differed by self-rated health, with a higher frequency in the self-rated good health group (58.0%) than in the self-rated poor health group (47.1%). Finally, the frequency differed by the number of chronic diseases, ranging from 57.7% for those having no chronic disease, 53.1% for those having one chronic disease, and 50.7% for those having two or more chronic diseases. These sociodemographic factors that differed between the “willing” and “unwilling” groups were included as covariables in the following analysis.

### 3.2. Contribution of PMT in Predicting the Willingness to Receive the Vaccine

A hierarchical regression model with the willingness (range 1–7) and PMT constructs (range 1–5 for each construct) as the continuous variables was performed to examine the relative contribution of PMT in predicting the willingness to obtain the COVID-19 vaccine. As shown in Table 3, age, gender, occupation, region, self-rated overall health, and the number of chronic diseases were entered as independent variables in the first block. PMT constructs, including perceived severity, perceived susceptibility, response efficacy, self-efficacy, and costs, were entered as independent variables in the second block. The willingness to receive the COVID-19 vaccine was entered as the dependent variable. The overall model explained a substantial and significant 26.6% (*p* < 0.001) of the variance in willingness to receive the COVID-19 vaccine. The first model with sociodemographic factors explained only 2% (*p* < 0.001) of the variance in willingness to receive the COVID-19 vaccine, which was mainly contributed by four sociodemographic factors: age (b = 0.007, *p* = 0.040), gender (women vs. men, b = −0.214, *p* = 0.001), region (rural vs. urban, b = 0.204, *p* = 0.038), and self-rated overall health (poor vs. good, b = −0.383, *p* < 0.001). After accounting for sociodemographic factors in the first model, the PTM model comprising perceived severity, perceived susceptibility, response efficacy, self-efficacy, and response costs explained an additional 24.6% (*p* < 0.001) of the variance in willingness to receive the COVID-19 vaccine, which was mainly contributed by three coping appraisal constructs: response effects (b = 0.685, *p* < 0.001), self-efficacy (b = 0.295, *p* < 0.001), and response costs (b = −0.403, *p* < 0.001).

### 3.3. Psychosocial Predictors of the Willingness to Receive the COVID-19 Vaccine

**Figure 1:**
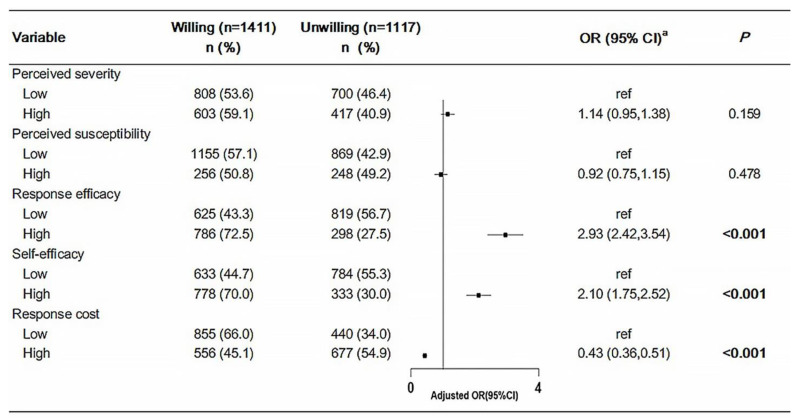
Multiple logistic regression for psychosocial factors predicting the willingness to receive the COVID-19 vaccine. (^a^ OR was adjusted for age, sex, occupation, region, self-rated overall health, the number of chronic diseases, and all the psychosocial factors).

Furthermore, follow-up logistic regressions were run with “willing” or “unwilling” to get the COVID-19 vaccination as the outcome to illustrate the psychosocial predictors of willingness to receive the COVID-19 vaccine. As shown in Figure 1, the multivariable logistic regression analysis was used to test the psychosocial factors associated with the willingness to receive the COVID-19 vaccine, adjusting for age, gender, occupation, region, self-rated overall health, number of chronic diseases, and psychosocial factors. Among the PMT constructs, response efficacy, self-efficacy, and response costs to the COVID-19 vaccination were identified as potential predictors for the willingness to receive the vaccination, and response efficacy was the strongest predictor. Respondents with a high level of response efficacy towards vaccination were 2.93 times more likely to be willing to receive the vaccination than were those with a low level of response efficacy (OR = 2.93, 95% CI: 2.42–3.54, *p* < 0.001). Compared with those with a low level of self-efficacy, respondents with a high level of self-efficacy had increased adjusted odds of willingness to have the vaccination (OR = 2.10, 95% CI: 1.75–2.52, *p* < 0.001). For response costs to the COVID-19 vaccination, high response costs may decrease the 57% tendency to be willing to receive the vaccination compared to the low response costs (OR = 0.43, 95% CI: 0.36–0.51, *p* < 0.001). 

### 3.4. Inspecting the Impact of Specific Items Influencing the Willingness to Obtain the COVID-19 Vaccine

**Figure 2:**
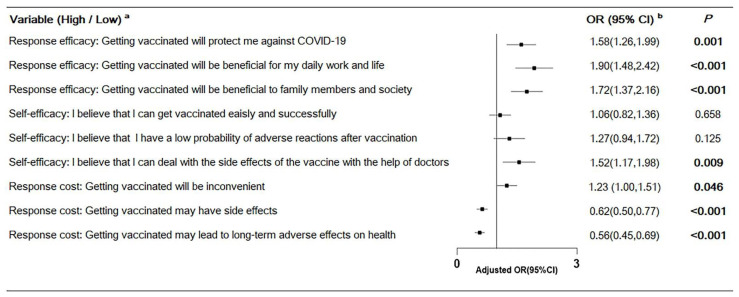
Multiple logistic regression for specific items predicting the willingness to receive the COVID-19 vaccine. (^a^ The perceived degree of each item was obtained (range 1–5), and the median of each item index was used for binary categorical classification (high/low level). ^b^ OR was adjusted for age, sex, occupation, region, self-rated overall health, the number of chronic diseases, and all the psychosocial factors).

To obtain an indication of the relative contribution of specific items to the significance of psychosocial constructs, further multivariable logistic regression analyses were run. As shown in Figure 2, predictions of willingness to receive the COVID-19 vaccine included a high level of response efficacy in protecting against COVID-19 (OR = 1.58, 95% CI: 1.26–1.99, *p* = 0.001), benefits for daily work and life (OR = 1.90, 95% CI: 1.48–2.42, *p* < 0.001), benefits for family members and society (OR = 1.72, 95% CI: 1.37–2.16, *p* < 0.001), a high level of self-efficacy in regard to dealing with the side effects of the vaccine with the help of doctors (OR = 1.52, 95% CI: 1.17–1.98, *p* = 0.009), and a high level of response cost in inconvenient situations (OR = 1.23, 95% CI: 1.00–1.51, *p* = 0.046). A high level of response cost of side effects (OR = 0.62, 95% CI: 0.50–0.77, *p* < 0.001) and long-term adverse effects on health (OR = 0.56, 95% CI: 0.45–0.69, *p* < 0.001) were predictions of unwillingness to receive the COVID-19 vaccine.

## 4. Discussion

The present study uses PMT to guide the selection of potential psychosocial factors influencing the Chinese public’s willingness to receive the COVID-19 vaccination when faced with the emergency use authorization release of the COVID-19 vaccine by the CNMPA. The results showed a 55.8% “willing” rate of getting vaccinated in the early stage of the COVID-19 vaccine approval for emergency use. Coping appraisals, including response efficacy, self-efficacy, and costs, had significant influences on one’s willingness to receive the COVID-19 vaccine; however, threat appraisals, including perceived severity and perceived susceptibility, had no effect on one’s willingness to receive the COVID-19 vaccine.

Importantly, our findings indicated that coping appraisals, including response efficacy, self-efficacy, and costs, had significant influences on the willingness to receive the COVID-19 vaccine, with response efficacy as the strongest psychosocial predictor. Respondents with a high level of response efficacy to the vaccination were 2.93 times more likely to be willing to receive the COVID-19 vaccine than those with a low level of self-efficacy. Consistent with our findings, response efficacy was found to be an important determinant of the HPV vaccination intentions among three target groups from the Canadian population [27]. In an earlier study of parents of middle school children in the Italian-speaking part of Switzerland, however, only the response (vaccination) efficacy was shown to be directly related to the parents’ intention to adhere to the combined measles, mumps, and rubella vaccination recommendations [28]. As expected, compared with a low level of self-efficacy, a high level of self-efficacy was significantly associated with the willingness to get vaccinated. Self-efficacy was also the construct in the HBM [29] and TPB [30] theatrical models. Guidry et al. [10] reported that scoring high on self-efficacy in regard to getting a COVID-19 vaccine is a predictor of willingness to take a COVID-19 vaccine both with and without emergency use authorization in the USA. This relationship of self-efficacy with the willingness to get the COVID-19 vaccine was further supported herein based on our findings in the Chinese population in the early stage of approval of the COVID-19 vaccine. Regarding response costs, such costs as a whole were found to be inversely associated with the willingness to get vaccinated. A recent review of studies on vaccination decisions that parents make for their children also found strong influences of the expectation of adverse vaccine effects and practical difficulties, which might be seen in context with self-efficacy [31,32].

We did not observe an association between threat appraisals, including perceived severity and perceived susceptibility, and willingness to receive the COVID-19 vaccine. These findings have also been indicated by several studies on vaccination intent/willingness. Gainforth et al. [27] reported that the perceived severity of HPV was not a determinant of HPV vaccination intentions among any of the three target groups from the Canadian population. Kim et al. [33] found that, during the second wave of the influenza outbreak in Arizona, the perceived likelihood of getting sick (cognitive element) was not strongly associated with preventive behaviors, whereas perceived concern (emotional element) was significantly associated with precautionary and preparatory behaviors. A systematic review showed good evidence for an association between vaccination and perceived susceptibility to the illness, but evidence for an association between perceived severity of an illness and vaccination was weak [31]. However, there are also opposite findings reporting the important impact of perceived severity and perceived susceptibility on vaccination intent/willingness. A survey study in China identified threat severity and expected vaccination risk as decisive effects regarding the intention to get vaccinated against the same H1N1 virus [34]. A systematic review of factors in getting vaccinated against H1N1 also found effects of threat severity [35]. Multiple factors, including different targeted populations, different vaccines, different infectious diseases (new or known), and different questionnaires, although located in the same construct, may contribute to these inconsistent findings. More importantly, two interpretations of our findings are offered. First, we are now in the middle of the pandemic, and the public’s attention has shifted from the COVID-19 disease itself to coping appraisals for vaccinations. Second, the Chinese government’s prevention and control measures are comprehensive and in-depth, and the control scope based on the detected new case of COVID-19 is very timely; therefore, people in China tend to perceive a low level of susceptibility to COVID-19, as evidenced by the fact that 80.0% (2024/2528) of the respondents herein reported having a low level of perceived susceptibility to COVID-19.

Looking more closely at the specific indications that affect the willingness to get the COVID-19 vaccine, we found that all three items, including the response efficacy for protecting against COVID-19, benefits for daily work and life, and benefits for family members and society, were predictors of the willingness to get the COVID-19 vaccine, thereby providing the pivotal role of response efficacy in the willingness to get vaccinated against emerging infectious diseases. For self-efficacy, only the variable “can deal with the side effects of the vaccines with the help of doctors” was a significant predictor. Of the three items located within response cost, the response cost of side effects and long-term adverse effects on the health of vaccination behavior were two important predictors of the unwillingness to get vaccinated. Consistently, parents who delayed and refused vaccines were shown to be less likely to believe that vaccines are safe [36,37]. Interestingly, we found a positive relationship between inconvenient response costs and willingness to get vaccinated, which may be due to a reverse causality because people who are willing to get vaccinated might prefer a convenient process.

Sociodemographic characteristic factors should also be given attention to in terms of health education and health promotion. Our findings suggested that people who are older, men, those living in rural areas, and those who have self-rated good health are more likely to be willing to receive the COVID-19 vaccine compared with those who are younger, women, and those living in urban areas (Table 2). The higher an individual’s age, the more willing the individual is to get vaccinated, which can be explained by the fact that older people are highly susceptible to COVID-19. In our study, 75.8% of the respondents were aged 18–39; therefore, we speculate that women are less likely to get vaccinated than men, probably because they worry that the vaccination will affect their future fertility. People living in rural areas are more likely to get vaccinated than those living in urban areas, which may be due to the relatively worse medical conditions found in rural areas compared with those found in urban areas and, in turn, more worries about medical treatment when infected with the COVID-19 virus. Furthermore, our results support the positive relationship of self-rated overall health with the willingness to get vaccinated, which may be due to the fear that those in poor health cannot afford the side effects of such a vaccination.

Limitations should be noted when interpreting the results of this study. First, the online survey was based on social media responses, which may skew the results toward younger, more educated, and urban people, thus affecting generalizability. Second, the survey was completed in a relatively short time period; thus, the results may not reflect the long-term effect of these identified influencing factors because the willingness to obtain the vaccine will increase with the increase in the number of vaccinated persons. Third, although self-rated measures are very convenient and common in some fields of media research [38], the measurements may not be accurate and stable enough. Fourth, this study relied on cross-sectional survey data to examine the relationships; therefore, the results of the analyses should be interpreted with care because causal relationships between the variables may exist. Finally, the information on vaccine safety and efficacy was not provided in the current survey; therefore, there may be differences in the knowledge of vaccine safety and efficacy among respondents. Future studies need to investigate the relationship between knowledge of the vaccine and vaccination willingness.

## 5. Conclusions

This study demonstrated the importance of coping appraisal in developing education and interventions to promote the vaccination willingness in the early stage of the emergency use authorization of the COVID-19 vaccine and to enhance public health outcomes during such a pandemic. Efforts to enhance the willingness to receive the COVID-19 vaccine can be specified as presenting more scientific evidence about vaccination benefits and explaining how to deal with possible side effects through mass media, enforcing more transparent supervision, and providing more convenient ways of vaccination.

## Figures and Tables

**Table 1:** Items used to assess the constructs and the factor analysis results of the protection motivation theory (PMT) factors of willingness to get the COVID-19 vaccine.

PMT Constructs	Questions	Assignment and Variable Processing	Mean (SD)	Factor Loading	Cronbach’s Alpha
**Threat appraisal**					
Perceived Severity	I feel that (a) COVID-19 is a serious infection that is harmful to my health. (b) it would be very painful to get COVID-19. (c) Getting infected with COVID-19 would seriously affect my family.	1 = Strongly disagree, 2 = Disagree, 3 = Neutral, 4 = Agree, 5 = Strongly agree. The median of the respondents’ averaged index (median = 4.33) was used for binary categorical classification (high/low level).	4.06 (0.89)	0.749 0.872 0.862	0.80
Perceived Susceptibility	(a) I am vulnerable to being infected with COVID-19. (b) People around me are vulnerable to being infected with COVID-19.	1 = Strongly disagree, 2 = Disagree, 3 = Neutral, 4 = Agree, 5 = Strongly agree. The median of the respondents’ averaged index (median = 2.00) was used for binary categorical classification (high/low level).	1.92 (0.83)	0.924 0.923	0.86
**Coping appraisal**					
Response efficacy	Being vaccinated against COVID-19 would be: (a) extremely effective in protecting me against COVID-19. (b) beneficial for my daily work and life. (c) beneficial to family members and society.	1 = Strongly disagree, 2 = Disagree, 3 = Neutral, 4 = Agree, 5 = Strongly agree. The median of the respondents’ averaged index (median = 4.00) was used for binary categorical classification (high/low level).	4.02 (0.76)	0.749 0.854 0.860	0.83
Self-efficacy	I believe that (a) I can get the COVID-19 vaccinated easily and successfully. (b) I have a low probability of adverse reactions after a vaccination. (c) I can deal with the side effects of the COVID-19 vaccine with the help of doctors.	1 = Strongly disagree, 2 = Disagree, 3 = Neutral, 4 = Agree, 5 = Strongly agree. The median of the respondents’ averaged index (median = 3.67) was used for binary categorical classification (high/low level).	3.63 (0.81)	0.770 0.809 0.794	0.73
Response cost	Receiving the COVID-19 vaccine: (a) would be inconvenient for me. (b) may have side effects (fever, pain, etc.). (c) may lead to long-term adverse effects on my health.	1 = Strongly disagree, 2 = Disagree, 3 = Neutral, 4 = Agree, 5 = Strongly agree. The median of the respondents’ averaged index (median = 3.00) was used for binary categorical classification (high/low level).	3.16 (0.89)	0.589 0.888 0.896	0.69

**Table 2:** Descriptive statistics of 2528 participants.

		Willingness to Get the COVID-19 Vaccine (*n* = 2528)		
Variables	Overall (2528) *n* (%)	Willing (*n* = 1411) *n* (%)	Unwilling (*n* = 1117) *n* (%)	*p*
**Demographics**				
Age (years)				0.033
18–29	926 (36.6)	514 (55.5)	412 (44.5)	
30–39	990 (39.2)	526 (53.1)	464 (46.9)	
40–49	410 (16.2)	247 (60.2)	163 (39.8)	
50–59	202 (8.0)	124 (61.4)	78 (38.6)	
Gender				0.001
Women	1484 (58.7)	788 (53.1)	696 (46.9)	
Men	1044(41.3)	623 (59.7)	421 (40.3)	
Marital status				0.419
Married	1658 (65.6)	935 (56.4)	723 (43.6)	
Not married	870(34.4)	476 (54.7)	394 (45.3)	
Educational attainment				0.443
High school degree and below	204 (8.1)	121 (59.3)	83 (40.7)	
Bachelor degree	1607 (63.6)	884 (55.0)	723 (45.0)	
Master’s degree and above	717 (28.4)	406 (56.6)	311 (43.4)	
Occupation				0.004
Medical staff	497 (19.7)	306 (61.6)	191 (38.4)	
Non-medical staff	2031(80.3)	1105(54.4)	926 (45.6)	
Region				0.045
Urban	2260 (89.4)	1246 (55.1)	1014 (44.9)	
Rural	268 (10.6)	165 (61.6)	103 (38.4)	
Self-rated overall health				<0.001
Good	2018 (79.8)	1171 (58.0)	847 (42.0)	
Poor	510 (20.2)	240 (47.1)	270 (52.9)	
Number of chronic diseases				0.028
0	1616 (63.9)	933 (57.7)	683 (42.3)	
1	638 (25.2)	339 (53.1)	299 (46.9)	
2 and above	274 (10.8)	139 (50.7)	135 (49.3)	

**Table 3:** Hierarchical regression model testing of PMT in COVID-19 vaccination willingness.

Independent Variables	*b (SE)*	*SE*	*β*	*t*	*p*	Δ*R^2^, F (x, y), p*
Block 1: Sociodemographic characteristic						
Age	0.007	0.004	0.042	2.058	0.040	ΔR^2^ = 0.020 F (x, y) = 8.733 (62,521), *p <* 0.001
Gender: women (Ref: men)	−0.214	0.062	−0.069	−3.456	0.001	
Occupation: non-medical staff (Ref: medical staff)	−0.054	0.077	−0.014	−0.706	0.480	
Region: rural (Ref: urban)	0.204	0.099	0.041	2.071	0.038	
Self-rated overall health: poor (Ref: good)	−0.383	0.078	−0.1	−4.893	<0.001	
Number of chronic diseases	−0.063	0.047	−0.028	−1.341	0.180	
Block 2: PMT constructs						
Perceived severity	−0.037	0.033	−0.022	−1.12	0.263	ΔR^2^ = 0.246 F (x, y) = 168.944 (52,516), *p* < 0.001
Perceived susceptibility	−0.15	0.032	−0.008	−0.469	0.639	
Response efficacy	0.685	0.041	0.338	16.634	<0.001	
Self-efficacy	0.295	0.038	0.155	7.87	<0.001	
Response cost	−0.403	0.032	−0.233	−12.621	<0.001	

Note. ΔR^2^ model = 0.266, *p* < 0.001. Dependent variable = vaccination willingness.

## Data Availability

The data that support the findings of this study are available from the school of public health, Fudan University, but restrictions apply to the availability of these data, which were used under a license for the current study, and, hence, are not publicly available. Data are, however, available from the authors upon reasonable request and with the permission of the school of public health, Fudan University.
